# BayMiR: inferring evidence for endogenous miRNA-induced gene repression from mRNA expression profiles

**DOI:** 10.1186/1471-2164-14-592

**Published:** 2013-08-30

**Authors:** Hossein Radfar, Willy Wong, Quaid Morris

**Affiliations:** 1 Department of Electrical and Computer Engineering, University of Toronto, Toronto, Ontario, Canada; 2 Department of Computer Science, University of Toronto, Toronto, Ontario, Canada; 3 Department of Molecular Genetics, University of Toronto, Toronto, Ontario, Canada; 4 Terrence Donnelly Centre for Cellular and Biomolecular Research, University of Toronto, Toronto, Ontario, Canada

## Abstract

**Background:**

Popular miRNA target prediction techniques use sequence features to determine the functional miRNA target sites. These techniques commonly ignore the cellular conditions in which miRNAs interact with their targets in vivo. Gene expression data are rich resources that can complement sequence features to take into account the context dependency of miRNAs.

**Results:**

We introduce BayMiR, a new computational method, that predicts the functionality of potential miRNA target sites using the activity level of the miRNAs inferred from genome-wide mRNA expression profiles. We also found that mRNA expression variation can be used as another predictor of functional miRNA targets. We benchmarked BayMiR, the expression variation, Cometa, and the TargetScan “context scores” on two tasks: predicting independently validated miRNA targets and predicting the decrease in mRNA abundance in miRNA overexpression assays. BayMiR performed better than all other methods in both benchmarks and, surprisingly, the variation index performed better than Cometa and some individual determinants of the TargetScan context scores. Furthermore, BayMiR predicted miRNA target sets are more consistently annotated with GO and KEGG terms than similar sized random subsets of genes with conserved miRNA seed regions. BayMiR gives higher scores to target sites residing near the poly(A) tail which strongly favors mRNA degradation using poly(A) shortening. Our work also suggests that modeling multiplicative interactions among miRNAs is important to predict endogenous mRNA targets.

**Conclusions:**

We develop a new computational method for predicting the target mRNAs of miRNAs. BayMiR applies a large number of mRNA expression profiles and successfully identifies the mRNA targets and miRNA activities without using miRNA expression data. The BayMiR package is publicly available and can be readily applied to any mRNA expression data sets.

## Background

MicroRNAs are short (21-25 nt) non-coding RNAs that repress the expression of their direct targets [1-4]. Primary miRNAs (pri-miRNAs) are transcribed from intra/intergenic genomic loci and cleaved by Drosha to form approximately 70-nt hairpin precursors (called pre-miRNAs) that are subsequently cleaved by the RNase III enzyme, Dicer, to generate miRNA duplexes [5]. One strand of the duplex, the mature miRNA, is loaded into the RNA-induced silencing complex (RISC) [6] and guides it to recognize mRNA targets through partial base pairing with the 3’ UTRs of targets [7].

The presence of target sites with perfect complementarity to the seed region of miRNAs is a strong predictor of targeting but perfect complementarity is neither sufficient nor necessary [7-10]. Many other determinants have been proposed to specify efficient mRNA-miRNA duplexes including: AU composition flanking target sites [8], thermodynamic stability of binding sites [11], evolutionary conservation of the seed [12-14], secondary structure accessibility [6,15-17], target-site abundance [18,19], seed-pairing stability [18], 3’ pairing contribution [8], loop in position 9-12 of miRNA-mRNA hybrids [10], and the binding location in the 3’ UTR [8,17]. Due to the limited number of validated miRNA targets, the exact specificity and sensitivity of current determinants are unclear [20-23]; however, estimates of precision of these determinants, alone or together, are typically reported to be about 50% at a sensitivity of 6-12% [24,25], suggesting that sequence-based prediction methods are not fully capturing miRNA target preferences.

In mammals, it is estimated that miRNAs primarily and dominantly repress the steady-state expression level of their targets [26-34]. Therefore, down-regulation of an mRNA’s expression when the miRNA is active is evidence of a functional target site on the gene *in vivo*. Although numerous methods have been introduced to incorporate mRNA and miRNA expression data into miRNA target predictions, existing methods either require paired miRNA-mRNA data [35-48], have only been tested in miRNA transfection assays [28,29,49], or do not consider the combinatorial impact of multiple miRNAs on mRNA expression [50,51].

In this paper, we introduce two new mRNA-miRNA scoring schemes by incorporating genome-wide measures of mRNA expression in target prediction. Neither of these scoring schemes requires miRNA expression data, so can be applied to vast amount of publicly available mRNA expression databases. The first scoring scheme identifies the impact of a miRNA in repressing an mRNA in presence of other targeting miRNAs, cellular activities, and under a wide range of endogenous conditions. This scheme (hereafter called the BayMiR score) is obtained using BayMiR, a sparse Bayesian linear regression model, in which the decrease in expression levels of an mRNA across different conditions is explained in terms of the activity of miRNAs that have conserved target site matches in the 3’ UTR of the transcript. BayMiR infers miRNA activity levels based on the expression profiles of its putative targets (predicted on the basis of conserved seed matches) and then it refines these target predictions using the regression model. We also found that expression variability is significantly higher among mRNAs with more miRNA target sites and, furthermore, that it can be used to identify more likely targets. Accordingly, we used the variance of gene expression levels across a wide range of samples including different cell types, cell lines, and disease/healthy tissues as another mRNA-miRNA scoring scheme. These scores are called “gene variation” index.

BayMiR analysis was conducted on 1,539 human miRNAs and the expression levels of 13,303 genes measured on 5,372 microarray experiments and predicts that approximately 60% of miRNA-mRNA duplexes with matched conserved targets sites have detectable down-regulation signal on gene expression. We evaluated and compared the efficacy of the proposed scores with eight TargetScan scores (a collection of most important sequence based features) as well as Cometa scores (an mRNA expression based miRNA target prediction method) using over-expression miRNAs experiments, validated targets, and GO and KEGG enrichment analysis. Using these benchmarks, we found the BayMiR scores consistently outperform both the sequence and expression scores and identify to what extent down-regulated genes on a global set of microarrays are under control of miRNAs.

## Results

### BayMiR method

**Figure 1:**
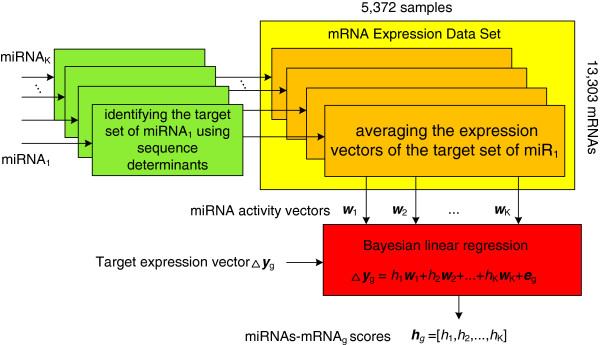
**BayMiR Method.** Flowchart of the BayMiR algorithm. For each miRNA, BayMiR first identifies the set of targets based on the presence of conserved complementary sites to the seed region of the miRNA in the 3’UTR of the target. Next, for each miRNA, BayMiR extracts the mRNA expression vectors associated with the selected targets from the mRNA gene expression data set, and averages them to obtain the miRNA activity vector. These miRNA activity vectors are used as regressors in a Bayesian linear regression model to explain the down-regulation in the expression level of the target. Finally, BayMiR infers scores (the regression coefficients) using a penalized likelihood method called elastic net regression. Each score indicates the strength of miRNA- mediated repression on the target genes.

BayMiR (Figure 1) calculates the degree to which mRNA down-regulation inferred from a large set of microarrays can be explained by inferred miRNA activity. BayMiR makes this prediction by integrating sequence and expression evidence. Because many targets are under the control of multiple miRNAs [20,46,52,53], BayMiR applies a linear model that relates the target expression vector (measured variable) to a weighted combination of the miRNA activity vectors (regressor variables). BayMiR infers the activity vector of a given miRNA by averaging the normalized expression vectors of its predicted mRNA targets based on sequence-based prediction methods. These miRNA activity vectors are then used as regressors in a Bayesian linear regression model of the “down-regulation” expression vector of each mRNA. The resulting regression coefficients of each miRNA are interpreted as the strength of miRNA-mediated repression of the target mRNA.

We also considered the variability in gene expression of a target mRNA as a determinant to distinguish functional and non-functional targets of a given miRNA. The gene variation index for each mRNA is computed as the variance of gene expression levels across all samples.

Each expression vector consists of the transcriptional abundance of the target in one of 392 biological samples collected from 5,372 microarray experiments. We determine the coefficients of the regression model using a penalized likelihood approach called elastic net regression [54] (see Methods) modified to assign only positive coefficients. By using this regression model, each sequence-predicted miRNA-mRNA interaction is assigned one coefficient; this coefficient represents how much the inferred activity profile of that miRNA contributes to predicting that mRNA’s “down-regulation” profile (see Methods) when considering the activity profiles of all other miRNAs predicted to target the mRNA. We call these coefficients “BayMiR scores” and interpret a zero BayMiR score as representing a lack of evidence in the expression data for regulation of the mRNA by that miRNA.

### BayMiR identifies highly repressed targets on miRNA over-expression assays

**Figure 2:**
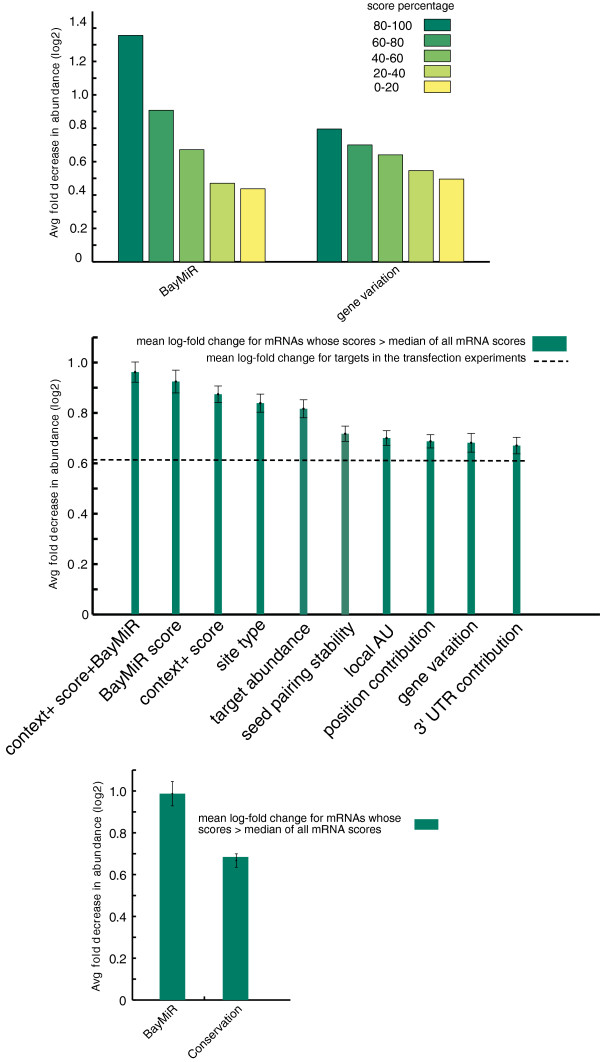
**BayMiR performance in the miRNA over-expression experiments.** (top) mRNAs in the over-expression miRNA assays are grouped into five bins based on their BayMiR and gene variation scores; the mean log-fold change of the mRNAs in each bin is plotted in as a bar. There are two groups of bars; the left- and right-hand groups correspond to BayMiR and gene variation, respectively. (middle) Comparing BayMiR and gene variation scores with seven sequence scores from TargetScan. Each bar represents the negative mean log-fold change for mRNAs whose scores are greater than the median of all mRNA scores for the selected determinant in the miRNA over-expression assays. The most left-hand group is obtained by combining the context+ scores with BayMiR scores. The dashed line shows the mean log-fold change for all targets in the miRNA over-expression assays (bottom) Comparing BayMiR scores with the conservation scores as measured by TargetScan. The conservation scores are given only for the targets with conserved target sites complementary to the seed regions of the conserved miRNA families. Error bars indicate 95% confidence intervals for the estimated means.

To evaluate whether the BayMiR scores reflect the strength of miRNA-mediated repression of mRNA targets, we measured the consistency between the BayMiR scores and relative down-regulation of targets in a set of miRNA over-expression experiments. One expects high scoring targets to be down-regulated more in miRNA over-expression experiments. We note that a similar metric has previously been used to evaluate the efficiency of TargetScan scores [8,18], and that this set of miRNA over-expression assays were not used in BayMiR to obtain the scores; thus, we are not influencing the results of our evaluation by either selecting bias metrics or by evaluating our model on the training data. We downloaded the data collected by Khan et. al [34] in which 23 miRNAs were transfected into seven different cell types and the log-fold change of the expression levels of mRNAs were measured. To examine that the degree to which our scores can predict the log-fold change of mRNAs in the miRNA over-expression arrays, for each score, we binned mRNAs into five bins based on their scores and computed the mean of mRNA log-fold changes in each bin. We observed that negative log-fold repression levels decrease consistently as scores decrease for both determinants (Figure 2.(top)). In total, 3,867 out of 10,125 mRNAs are down-regulated in the miRNAs over-expression experiments. We then asked if our scoring schemes can detect repressed targets better than the individual components of the TargetScan context score [8]. When comparing negative mean log-fold changes for messages whose scores were greater than the median score for the corresponding miRNA, BayMiR scores outperforms all TargetScan scores, even the context+score which is a combination of all individual TargetScan scores (Figure 2.(middle)). In addition, when we combined BayMiR scores and the TargetScan context+score the performance further improved (Wilcoxon-Mann-Whitney test: *P* < 0.001), indicating that BayMiR can augment the TargetScan scoring system to further improve the performance. Target site conservation is another scoring scheme used by TargetScan, so we also compared BayMiR scores with conservation scores for all conserved target sites of all conserved miRNA families and found similar improvements (Figure 2.(bottom)). Our analysis also shows that the gene variation score was a better predictor of log-fold change than seed pairing stability, relative location of seed match in the 3’ UTR, and target abundance; however, it is worse than the other components of the context score on this assay (Figure 2(middle)).

#### High-scoring BayMiR targets are enriched for validated targets

To test whether the set of experimentally validated targets are enriched among high-scoring BayMiR targets, we measured the significance of overlap between the targets with scores greater than the median and the experimentally validated targets retrieved from TarBase [55]. Enrichment using the hyper-geometric test showed that the validated targets are enriched in the sets of high-scoring genes both for BayMiR and gene variation predicted targets, *P* < 10^-5^ and *P* < 10^-4^ respectively. A cumulative distribution analysis is also shown in Additional file 1: Figure S1. Number of TarBase validated human targets at mRNA level is 491; number of validated targets with conserved target site is 279 and BayMiR predicts 203 of these conserved validated targets (72.8%). Together these observations support that the hypothesis that repressed targets under the endogenous conditions are more likely to be functional targets.

#### BayMiR predicts miRNA-induced repression better than Cometa

**Figure 3:**
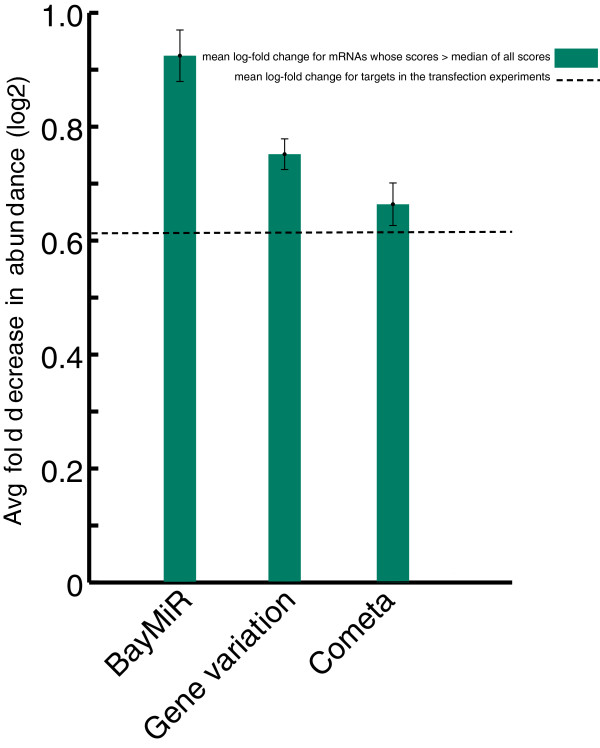
**Comparing BayMiR and Cometa.** BayMiR high scoring targets are more down-regulated in miRNA over-expression assays than Cometa high scoring targets. Each bar represents the mean of negative log-fold change after miRNA over-expression for genes with scores greater than median.

Next, we used the same evaluation strategy to compare BayMiR scores with an mRNA-miRNA scoring method which also uses large-scale gene expression data. Recently, Gennarino et al. [50] showed that the target set of a miRNA tend to be co-expressed and based on this property they proposed Cometa, a computational method that scores each sequence-based miRNA target prediction based on how correlated it is with other predicted targets of the miRNA. Examining the down-regulated targets on the miRNA over-expression assays shows that negative mean log-fold expression changes for targets selected by our scoring schemes are significantly higher than those selected by Cometa scores (*P* < 10^-40^, Additional file 2: Figure S2). Moreover, our methods’ high scoring targets are significantly more down-regulated compared to Cometa high scoring targets (*P* < 10^-60^ Figure 3) on the over-expression assays. Although Cometa targets are also enriched for validated targets, this enrichment is smaller than BayMiR scoring targets (*P* < 0.01 v.s. *P* < 10^-5^).

#### BayMiR target sets have more consistent GO-BP and KEGG annotations

Many miRNAs participate in the coordinate regulation of biological processes [56]; as such, we should expect that, in general, better target prediction methods would generate miRNA target sets that have higher enrichment [57]. To test whether BayMiR predicted targets are more consistently annotated with GO and KEGG terms than TargetScan targets, we used Fisher’s exact test with an FDR multiple test correction (see Methods) to score the enrichment of 1,233 GO-BP terms and 259 KEGG pathways within the target sets of each of 1,264 miRNA families. We found a nearly three-fold increase in enriched terms and pathways (*FDR* < 0.1) within BayMiR-predicted target sets compared to equally-sized random subsets of TargetScan (31,976 vs 11,890, *P* < 10^-200^).

Examination of the enriched GO-BP terms and KEGG pathways revealed a wide diversity of biological processes regulated by miRNAs (Additional file 3: Table S1, *FDR* < 0.1 and Additional file 4: Table S2, *FDR* < 0.1). We found that 35 % of miRNAs that have BayMiR target sets are enriched for the GO term “regulation of expression” suggesting that miRNAs have substantial influence in gene regulation through their control of other gene regulators.

We also searched for miRNAs with known functions among the miRNAs enriched in our pathway analysis. A list of miRNAs with experimentally supported functions among their enriched pathways are given in Additional file 5: Table S3. Notably the miR-17 family is frequently seen in the list. This family has been extensively studied and shown to play an important role in many cancer-related processes and pathways [58,59], and references in Additional file 5: Table S3.

**Figure 4:**
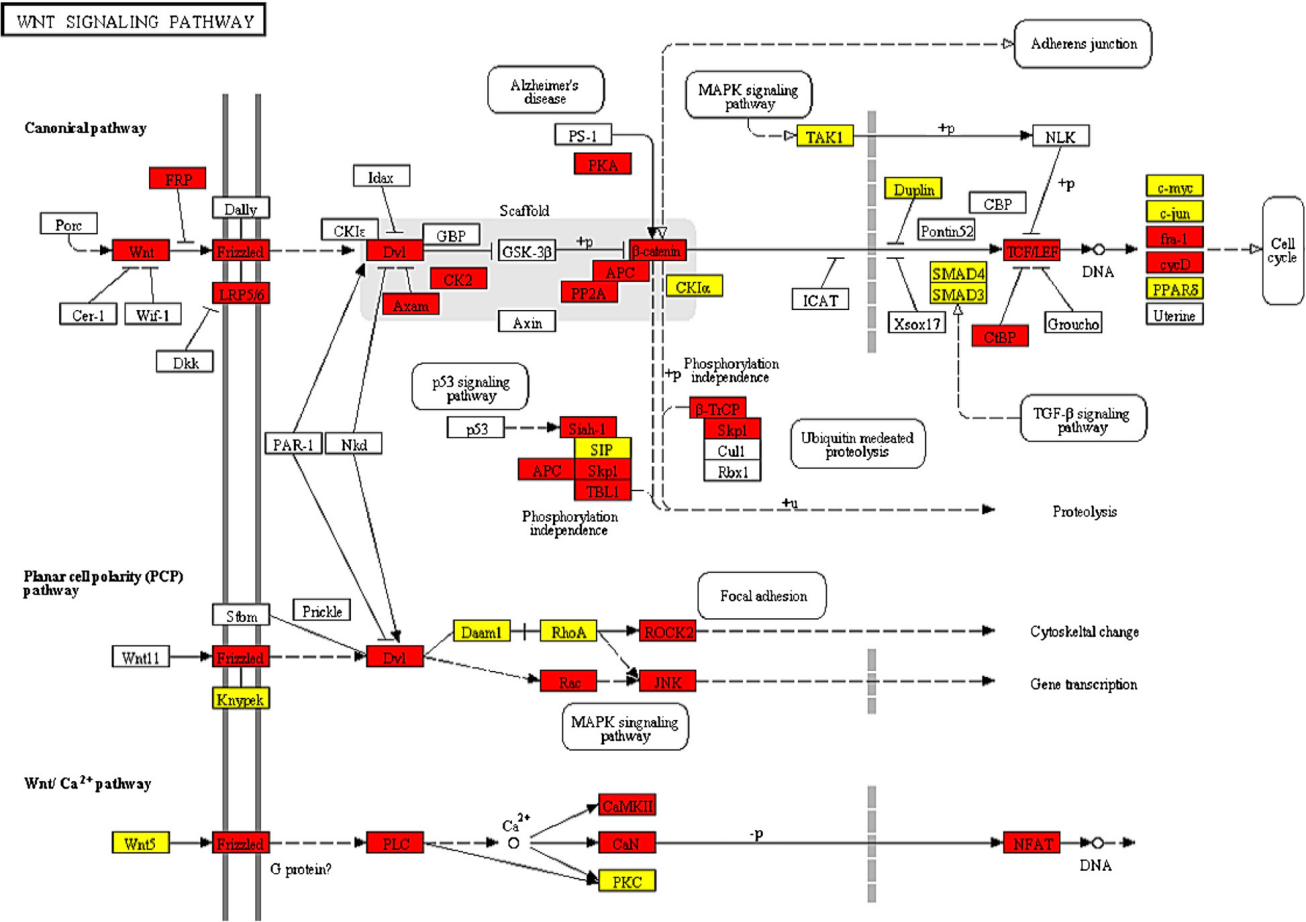
**WNT signaling pathway.** WNT signaling pathway: 32 targets of 5 miRNAs are involved in the pathway (red boxes). 14 mRNAs are targeted by the remaining miRNAs are colored in yellow; and 23 mRNAs involved in the pathway were excluded from the BayMiR target list since their expression variabilities across arrays were very low (white boxes). The miRNA family IDs: miR-518a-5p/520d-5p/524-5p,miR-556-3p,miR-4514/4692,miR-548aeajamx,miR-135ab/135a-5p;.

**Figure 5:**
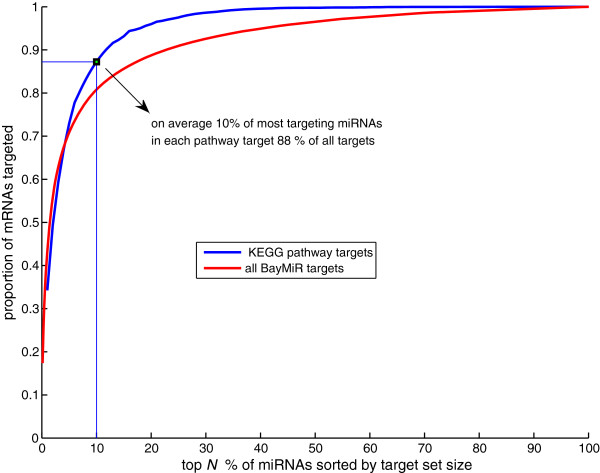
**miRNA targeting.** A small percent of all targeting miRNAs collectively target a large portion of miRNA targets. The figure shows two cumulative distributions. (red) Proportion of set of all mRNAs with BayMiR targets that are covered by union of the target sets of the top *N%* miRNAs (sorted by number of targets) where *N* increases along the x-axis. (blue) Average of cumulative distributions for all enriched KEGG pathways (n = 108), where each distribution was created as per the red line but restricted to targeted mRNA associated with the KEGG pathways, and their targeting miRNAs.

When we examined the mRNAs in KEGG pathways targeted by miRNAs, we found that although there are extensive co-regulation of mRNAs by multiple miRNAs, a handful of miRNAs appeared to be responsible for most of the regulation. For example, in the WNT signaling pathway, five miRNAs target 32 out of 46 genes predicted to be targeted by any of the 45 miRNAs with targets in this pathway (Figure 4). Similarly, the 106 genes in “Pathways in cancer” are targeted by 83 miRNAs but only 10 of these miRNAs collectively target more than 75% these genes (Additional file 6: Figure S3). Although some of this consolidation of targeting can be explained with a large variability in number of mRNA targets per miRNA, there is significantly more consolidation than we would expect by chance (Figure 5, *P* < 10^-19^) These observations suggest that important miRNA regulators of specific biological processes can be identified *in silico* through gene set enrichment analysis of BayMiR target sets.

### miRNA activity and expression profiles are significantly correlated

To test if miRNA activities obtained using the BayMiR procedure are correlated with the miRNA expression profiles, we downloaded the miRNA expression data from the mimiRNA repository [60] and computed the correlation between matched activity and expression vectors. After excluding miRNA expression data that are not consistent across multiple resources (according to *P* > 0.05 reported in the mimiRNA resource) and mapping the biological samples of the miRNA expression data to our biological groups we obtained paired matches for 48 miRNAs. Interestingly, we found that 96% of the pairs (46 out 48) have the Pearson correlation coefficients greater than 0.35 compared to 4% positive correlation obtained from a similar analysis but with the permuted activity vectors (*P* < 0.05 and Additional file 7: Table S4). This correlation analysis shows that miRNA activities inferred from the mean of inverse expression of their targets are highly correlated with expression data for those miRNAs.

### mRNAs harboring miRNA target sites near the both ends of the 3’ UTR have higher endogenous down-regulation signals

**Figure 6:**
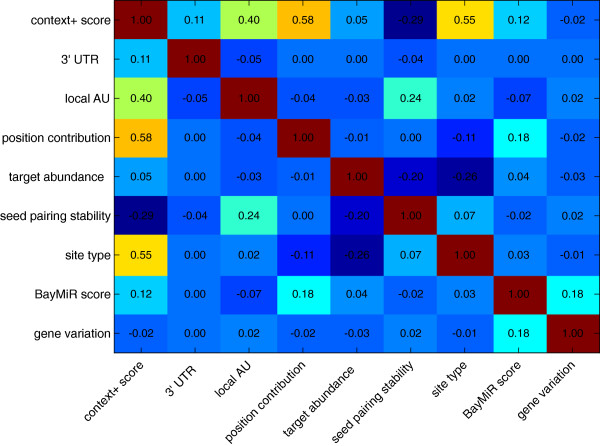
**Correlation between different determinants.** The heat map shows the Pearson correlation coefficients between each pair of nine determinants. A “0.00” correlation is shown for correlation coefficients that are not significantly different from 0 (*P* > 0.05).

To investigate any association between endogenous target repression scores provided by BayMiR and sequence and gene variation determinants, we measured the correlation between the scores of all paired determinants(Figure 6). The heat map shows that BayMiR scores correlate most highly with the position contribution scores. In addition, when we ranked all mRNA-miRNA pairs based on their BayMiR scores, the top 50 percentile of the ranked list have higher position contribution scores than the bottom 50 percentile (*P* < 10^-200^, Wilcoxon-Mann-Whitney test and Additional file 8: Figure S7). The position contribution scores provide estimate of expected repression in terms of the distance of targets sites from the both end of the 3’ UTR; target sites near to the ORF or the poly(A) tail are more effective [8] and more conserved than those in the middle of the 3’ UTR [12]. To further investigate this, we located 1,567,294 conserved target sites matched to the seed region of 1,032 miRNAs on the 3’ UTR of 17,840 mRNAs. The start position of each target site was divided by the length of the 3’ UTR to obtain the relative position of miRNAs on the 3’ UTRs, denoted by 0 < *L*_*miRNA*_ < 1. We found that target sites located on the both end of 3’ UTRs (*L*_*miRNA*_ < 0.25 or *L*_*miRNA*_ > 0.75) are assigned higher BayMiR scores than those on the middle (*P* < 10^-200^, Wilcoxon-Mann-Whitney test). Furthermore, we found that target sites located in the terminus close to the poly(A) tail (*L*_*miRNA*_ > 0.75) are assigned higher BayMiR scores than to those located on the other terminus (*L*_*miRNA*_ < 0.25, *P* < 10^-5^, Wilcoxon-Mann-Whitney test). Poly(A) shortening is known as one of the mechanisms of mRNA degradation; this mechanism strongly favors the preference of miRNA target sites near the end of 3’UTR close to the poly(A) tail to recruits mRNA deadenylase complexes [61]. Together these lines of evidence underline the importance of target site position in miRNA targeting.

BayMiR scores are also highly correlated with gene variation scores suggesting that mRNAs with high expression variability are under selective pressure to be miRNA targets.

## Discussion

Large-scale mRNA expression profiling datasets provide a rich resource to study the regulatory impact of miRNAs. Here, we showed that the impact of miRNAs on targets is detectable in normal tissue and unperturbed cell line data. Given a list of miRNAs with partial complementarity to a particular mRNA, our computational technique, BayMiR, scores the relative regulatory impact of the miRNA among other predicting targeting miRNAs. We showed that BayMiR estimates of miRNA regulatory impact better reflect independent measures of this impact than the TargetScan context scores; furthermore, we showed that the context scores and BayMiR can be combined to generate even better estimates. We also demonstrated that the miRNA activity vectors that we infer from mRNA expresssion data are well-correlated with the measured expression levels of these miRNAs.

BayMiR has several features that make it particularly useful for estimating the potential regulatory impact of a miRNA. BayMiR models the combinatorial effect of multiple regulatory miRNAs on a single target which is critical, as most mRNAs are likely to be targeted by multiple miRNAs (Additional file 9: Figure S4). BayMiR is fast; its runtime is less than a minute in the current version, so is easily applied to a subset of or all available gene expression data. Because BayMiR estimates the activity of miRNAs based on mRNA expression data, there is no need for matching miRNA expression profiles. As such, BayMiR predictions can be easily extended when new miRNAs are found and the current version of BayMiR incorporates all miRNAs retrieved from the latest release of miRBase (v.19).

Combinatorial regulation by multiple miRNAs has been described for particular mRNAs [8,62] and is likely to play a large role in mRNA expression regulation [46]. Indeed, human 3’ UTRs contain conserved seed matches for on average 33 of miRNAs (median = 16) (Additional file 9: Figure S4). This combinatorial regulation may explain the observations that inverse correlation under endogenous condition between miRNA and mRNA expression does not provide strong and consistent evidence of targeting [60,63] and that the impact of miRNA regulation on mRNA levels can only be seen within the context of other miRNA regulations [46,63]. Additional file 10: Figure S5 shows a toy example where combinatorial regulation masks inverse correlation between miRNA regulators and their targets.

There are a large number of other methods [49-51,63-72] that infer either miRNA activity or predict miRNA targets based on the expression levels of their sequence-predicted targets, however, no method both infers miRNA activity and predicts miRNA targets while considering the impact of other miRNAs. For example, Cometa attempts to predict miRNA targets, by identifying tight, co-expressed clusters of sequence-predicted targets [50]; however it doesn’t account for combinatorial regulation by multiple miRNAs and provides no estimate of miRNA activity. Other methods such as Sylamer [49], and a number of web-based applications [66-68], identify miRNA seed regions that significantly enriched in the 3’ UTRs of down-regulated transcripts as a way of assessing miRNA activity level in a tissue. However, the performance of Sylamer when applied to endogenous gene expression data is unclear. In addition, it does not take into account multiple targeting effect of miRNAs and has not been used to score the individual miRNA-mRNA pairs. Other methods use paired miRNA-mRNA expression patterns to augment sequence-based target prediction [35-48]. These methods typically require paired miRNA and mRNA measurements in a large number of samples to generate reliable predictions. This type of paired expression data is however rare and unavailable for some miRNAs [73]. On the other hand, there is very large amount of mRNA expression data available for BayMiR. Two intronic miRNA target prediction methods, InMiR and Hoctar [51,63] predict the intronic miRNA targets using the expression levels of their host genes, and subsequently can also incorporate large mRNA expression data. However, these methods can only be applied to intronic miRNAs and only to those miRNAs whose host gene expression is a good surrogate for their activity. Many host gene expression levels are not good surrogates [63,74-76].

Our analysis also reveals that mRNAs with more target sites have higher expression variation when compared to a random subset of genes, and expression variance consistently increases as number of target sites do (*P* < 10^-33^, Additional file 11: Figure S6). These observations suggest that mRNAs with highly variable expression levels are much more likely to be regulated by miRNAs; our finding is consistent with recent reports that genes regulated by miRNAs have higher expression variability at among humans and between human and other primate species [77].

miRNA transfection experiments have suggested that the degree of mRNA repression induced by two seeds is equivalent to the product of repression induced by the seeds individually [8]. We have observed a similar effect. The version of BayMiR described here implicitly assumes multiplicative interactions because it log-transforms the mRNA expression levels before performing regression. Applying BayMiR to non-transformed expression levels assumes additive interactions and this version of BayMiR performs much worse in our benchmarks (data not shown).

In this paper, we introduced BayMiR and demonstrated its merits when compared to two the state-of-the-art miRNA computational prediction methods. BayMiR applies a more relevant biological model and uses a large collection gene expression data to decipher the impact of miRNAs on gene expression data. We measured this impact in terms of endogenous target repression scores for about half a million miRNA-mRNA duplexes. This new scoring strategy can be used alone or along with other sequence determinants to predict functional miRNA-mRNA interactions.

## Methods

### BayMiR model

BayMiR applies the following linear model to relate the changes in the log-transformed expression level of mRNAs to the activity level of miRNAs:

$$\underset{M\times 1}{\Delta y^{i}}=\underset{M\times K}{W}\underset{K\times 1}{h^{i}}+\underset{M\times 1}{\epsilon }$$

where $\Delta y^{i}\in R^{M}$ denote the change in the expression level of the *i*th mRNA measured across *M* samples and is obtained by subtracting the mean from **y**^*i*^; **W** = [ *w*_*m*,*k*_]_*M*×*K*_ denote the activity levels of *K* miRNAs across *M* samples, and each element of $h^{i}\in R^{+K}$ represents the contribution of the corresponding miRNA in down-regulating the expression of the *i*th mRNA; ***ϵ*** models error. In our problem *K* = 1,252; *M* = 369 and *i* = 1,… 13,000.

In this linear equation, *Δ***y**^*i*^ and **W** and are observed; **h**^*i*^ is the desired unknown variable. BayMiR infers **h** by maximizing its posterior probability of **h** given *Δ***y** and **W**:

$$\hat{h}=\arg\max\log p(h|\Delta y,W).$$

This inference problem can be written in form of a penalized linear regression optimization given by:

(1) $$\begin{array}{ll} \hat{h}=\arg\min & \underset{m}{\sum }{\left(\Delta y_{m}-w_{m,:}h\right)}^{2}+\lambda_{1}\underset{k}{\sum }h_{k}\\ & +\lambda_{2}\underset{k}{\sum }h_{k}^{2}\;\;\;\text{subject to:}\;\;\;h_{k}\geq 0\;\forall k \end{array}$$

where *λ*_*i*_s are two tuning parameters and **w**_*m*,:_ is a row vector representing the expression activity of miRNAs in the *m*th sample. We solved this optimization using the coordinate-descent method [54] in which, the objective function is partially optimized with respect to each individual coefficient in an iterative manner given by

(2) $$h_{j}=\frac{S\left(\sum_{m=1}^{M}(\Delta y_{m}-\sum_{k\neq j}^{K}w_{m,k}^{n}h_{k})w_{\text{mj}}^{n},\lambda_{1}\right)}{\sum_{m=1}^{M}w_{\text{mj}}^{n2}+\lambda_{2}}$$

where *S*(*x*,*t*) is the soft threshold operator defined as *sign*(*x*)(|*x*| − *t*)_+_ where (*y*)_+_ = 0 if *y* < 0 and (*y*)_+_ = *y* if *y* ≥ 0 [78].

Since miRNA and target mRNA expression data are anti-correlated [79], for each miRNA, BayMiR uses the negative mean of target expression levels as an estimate of the activity level of the miRNA as follows:

(3) $$\begin{array}{ll} w_{k}= & -\frac{1}{N_{k}}\overset{N_{k}}{\underset{i=1}{\sum }}y_{i}\;\;\;\text{where}\\ & N_{k}:\;\;\text{number of target genes for }k\text{th miRNA} \end{array}$$

and then each activity vector is normalized $w_{k}\leftarrow \frac{w_{k}}{∥w_{k}∥}$. As such, the activity of the miRNA will be deemed to be positive when its sequence-predicted targets are below their mean expression level. BayMiR considers a gene as a potential target of a miRNA if there is a complementary conserved match sites to the seed region of the miRNA.

### Processing mRNA expression data

The mRNA expression data were downloaded from the EMBL-EBI repository [80], available at http://www.ebi.ac.uk/gxa/experiment/E-MTAB-62. The data consists of 5,372 samples profiled on HG-U133A array platforms; As described in [80], the data were normalized and manually labeled into 369 biological groups covering a wide range of healthy/cancer tissues, conditions, and cell lines. We did the following processing on the retrieved expression data; all probe sets with no gene symbols were excluded. The samples belonging to each biological groups were averaged—the samples within one biological group are highly correlated (*ρ* > 0.85). An upper/lower threshold defined by *l*_*th*_ = *Q*_2_ − 1.5(*Q*4_-_*Q*_2_) and *u*_*th*_ = *Q*_4_ + 1.5(*Q*_4_ − *Q*_2_) respectively, when *Q*_2_ and *Q*_4_ represent the second and forth quartiles, were specified to detect and modify the extreme outliers. The outliers were then replaced with *l*_*th*_ or *u*_*th*_. The gene symbol list in both expression and sequence datasets were updated based on the latest release of the HUGO Gene Nomenclature Committee (HGNC) (Feb.2012) to have consistent gene symbols.

### MiRNA-mRNA interaction analysis

We downloaded the list of 19,055 protein coding gene symbols from HGNC database and the list of 1,537 miRNA IDs from MiRbase V.19. We then built seven 19,055×1,532 binary connectivity matrices based on the mRNA-miRNA interactions given by: Targetscan V6.1, [7] and TarBase [55]. All miRNAs are grouped into 1,251 miRNA families as defined by TargetScan—miRNAs sharing the same seed region. Conserved target sites are also retrieved from the TargetScan repository.

### Enrichment analysis

Gene ontology biological process (GO-BP) annotations were downloaded from the Gene Ontology Website on April 15th 2012. The file contains 14,000 annotations for 15,000 genes. The enrichment analysis was performed using Fisher Exact test. The test was performed on BayMiR predicted targets of each of miRNA families. The enrichment pvalues were corrected using Benjamini-Hochberg test [81] and a FDR cutoff equal to 0.1 was chosen to selected significant enrichment categories. The KEGG enrichment analysis carried out in a similar manner; The list of 253 KEGG human pathways were with associated genes downloaded from http://www.genome.jp/kegg/; Fisher exact test was used to find enriched pathways for BayMiR targets of all miRNA families.

### Availability of BayMiR and supporting data

The code for BayMiR is available at http://morrislab.med.utoronto.ca/BayMiR. package includes scripts and instructions to re-generate BayMiR scores from the “E-MTAB-62” file and sequence information, however, a pre-computed version of the BayMiR scores are also uploaded.

## Conclusions

We developed BayMiR, a new computational method for predicting the target mRNAs of miRNAs. BayMiR applies a large number of mRNA expression profiles and successfully identifies mRNA targets and miRNA activities without using miRNA expression data. We also showed that gene expression variability can be used to predict miRNA targets. Our analysis revealed the importance of miRNA target sites at 3’ UTR near to the poly (A) tails. The BayMiR package is publicly available and can be applied to any mRNA expression datasets.

## Competing interests

The authors declare that they have no competing interests.

## Authors’ contributions

Conceived and designed the experiments: MHR QM WW. Performed the experiments: MHR. Analyzed the data: MHR QM. Wrote the paper: MHR QM. All authors read and approved the final manuscript.

## Supplementary Material

Additional file 1 **Figure S1.** Cumulative distribution of scores for the validated targets. Validated targets are assigned higher BayMiR scores and gene variation scores compared to the other putative targets. Shown are the cumulative distributions of BayMiR (left plot) and gene variation scores (right plot) scores for validated targets (blue) and all putative targets (red). Click here for file

Additional file 2 **Figure S2.** Comparing BayMiR and Cometa. BayMiR high scoring targets are more down-regulated in miRNA over-expression assays than Cometa high scoring targets. The cumulative distribution of log-fold change for high-scoring mRNAs; blue, red, and black represent graphs associated with BayMiR, gene variation, and Cometa. Click here for file

Additional file 3 **Table S1.** Excel file. Enriched GO-BP terms Click here for file

Additional file 4 **Table S2.** Excel file. Enriched KEGG terms Click here for file

Additional file 5 **Table S3.** Validated KEGG pathways. List of miRNAs with proposed functions found in our enriched KEGG list; the third column gives the Pubmed IDs of the references. Click here for file

Additional file 6 **Figure S3.** KEGG “Pathways in cancer”: 68 targets of 10 miRNAs are involved in the pathway (red boxes). 38 genes targeted by the other miRNAs are colored in yellow; and 62 genes involved in the pathway were excluded from the BayMiR target list since their expression variabilities across arrays were very low (white boxes). The miRNA family IDs: miR-17/17-5p/20ab/20b-5p/93/106ab/427/518a-3p/519d,miR-548ah/3609,miR-4729,miR-203,miR-548p,miR-3647-3p,miR-300/381/539-3p,miR-142-5p,miR-545,miR-125a-5p/125b-5p/351/670/4319’. Click here for file

Additional file 7 **Table S4.** Excel file: miRNA expression data retrieved from the mimiRNA repository. Click here for file

Additional file 8 **Figure S7.** Blue: the position contribution scores of miRNA-mRNA pairs whose BayMiR scores  > *median*_*B* *a* *y* *M* *i* *R* *s* *c* *o* *r* *e* *s*_. Red: the position contribution scores of miRNA-mRNA pairs whose BayMiR scores  < *median*_*B* *a* *y* *M* *i* *R* *s* *c* *o* *r* *e* *s*_. Click here for file

Additional file 9 **Figure S4.** The 3' UTR of mRNAs harbor many conserved seed matches. Shown is the cumulative distribution of number of seed matches in the 3'UTR of 14,816 mRNA transcripts with at least one miRNA seed match. Click here for file

Additional file 10 **Figure S5.** Example of combinatorial regulation masking inverse correlation. Shown in green is the expression level of a target gene and in red the expression levels of three targeting miRNAs. The negative correlation of each individual miRNAs with the target is insignificant, but when considered together they explain perfectly the down-regulation impact of miRNAs. Click here for file

Additional file 11 **Figure S6.** Gene expression variability increases as the number of target sites increases in the 3’ UTR of genes. (top) miRNA targets have high expression variation. (bottom) Red and blue demonstrate the cumulative distributions of genes whose variance is larger than median and 75^th^ percentile, respectively. Dark: cumulative distribution of variances corresponding to all genes. Click here for file
