# Supplementary figures and images for: BayMiR: inferring evidence for endogenous miRNA-induced gene repression from mRNA expression profiles
Source: BMC Genomics. 2013 Aug 30;14:592. doi: 10.1186/1471-2164-14-592 (PMC3933272; doi:10.1186/1471-2164-14-592)

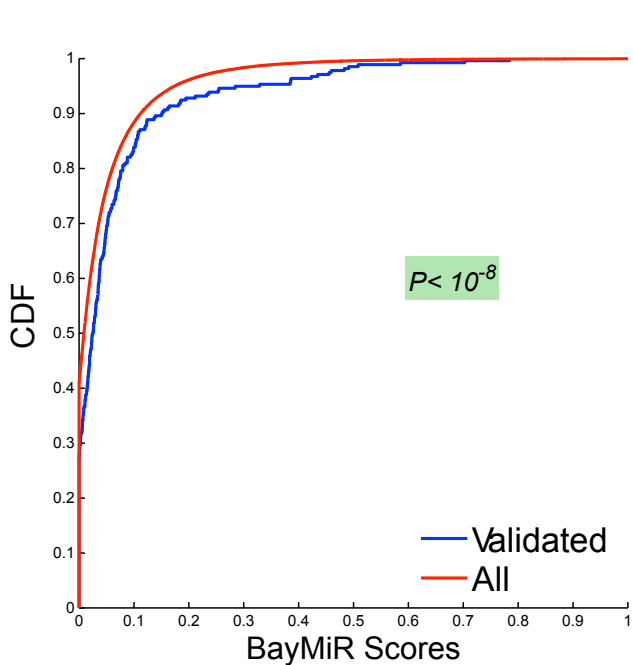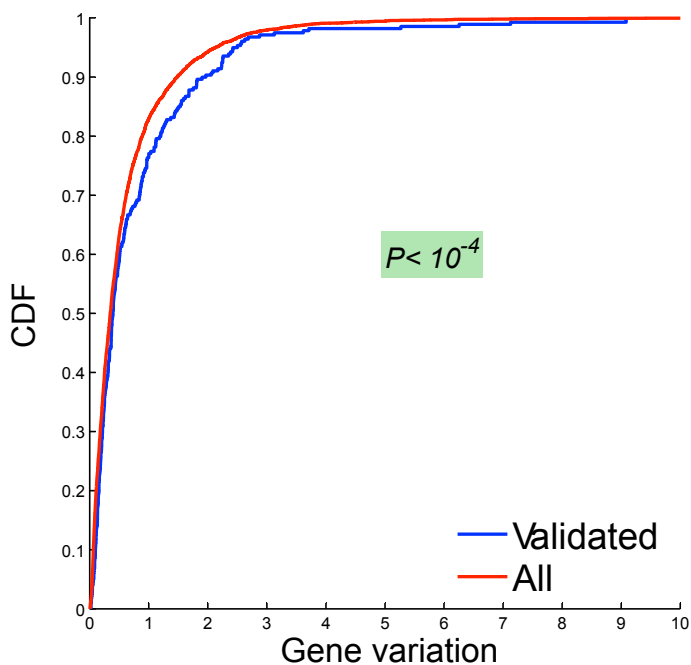

Supplement: Additional file 1 — Figure S1. Cumulative distribution of scores for the validated targets. Validated targets are assigned higher BayMiR scores and gene variation scores compared to the other putative targets. Shown are the cumulative distributions of BayMiR (left plot) and gene variation scores (right plot) scores for validated targets (blue) and all putative targets (red). [file 1471-2164-14-592-S1.pdf]

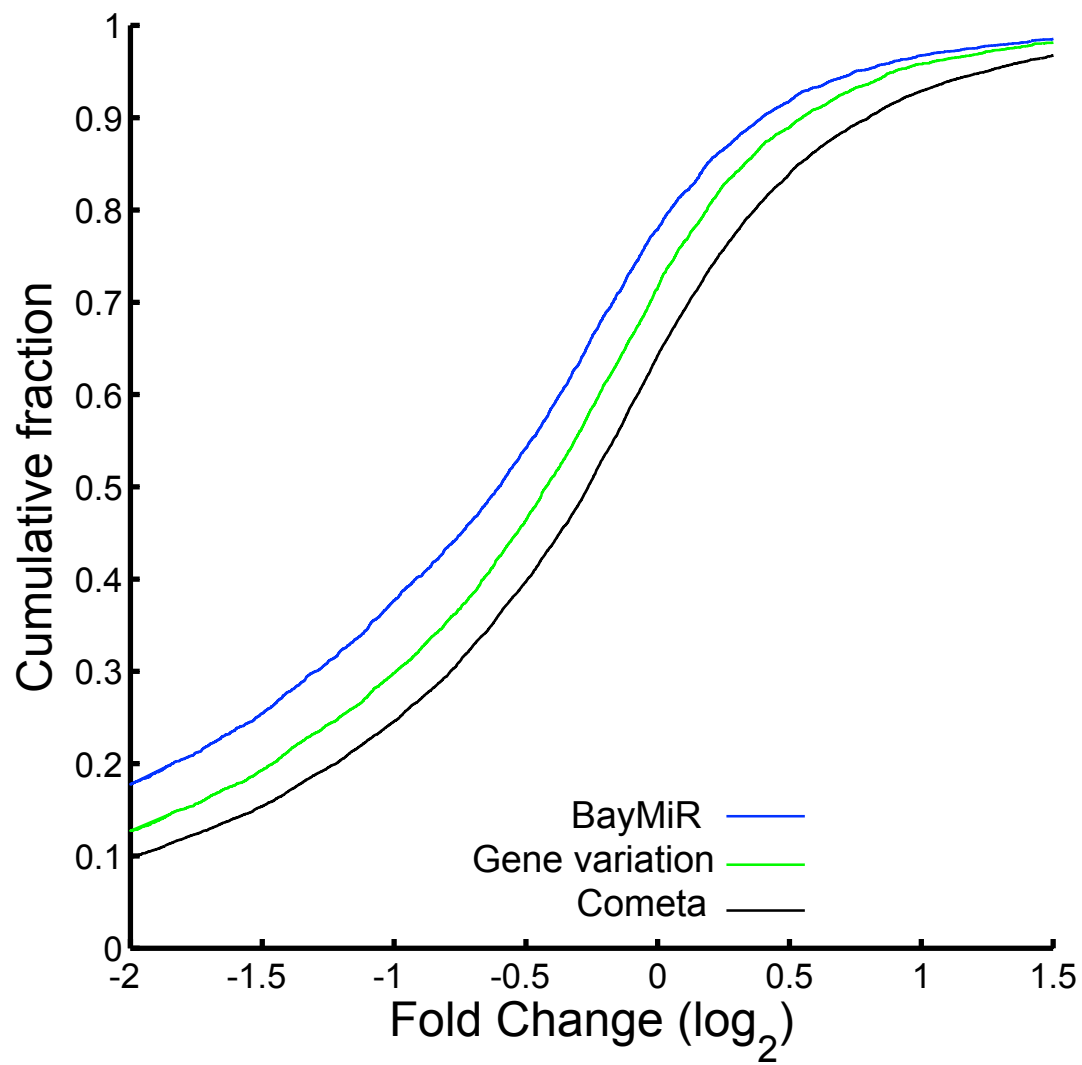

Supplement: Additional file 2 — Figure S2. Comparing BayMiR and Cometa. BayMiR high scoring targets are more down-regulated in miRNA over-expression assays than Cometa high scoring targets. The cumulative distribution of log-fold change for high-scoring mRNAs; blue, red, and black represent graphs associated with BayMiR, gene variation, and Cometa. [file 1471-2164-14-592-S2.pdf]

## PATHWAYS IN CANCER

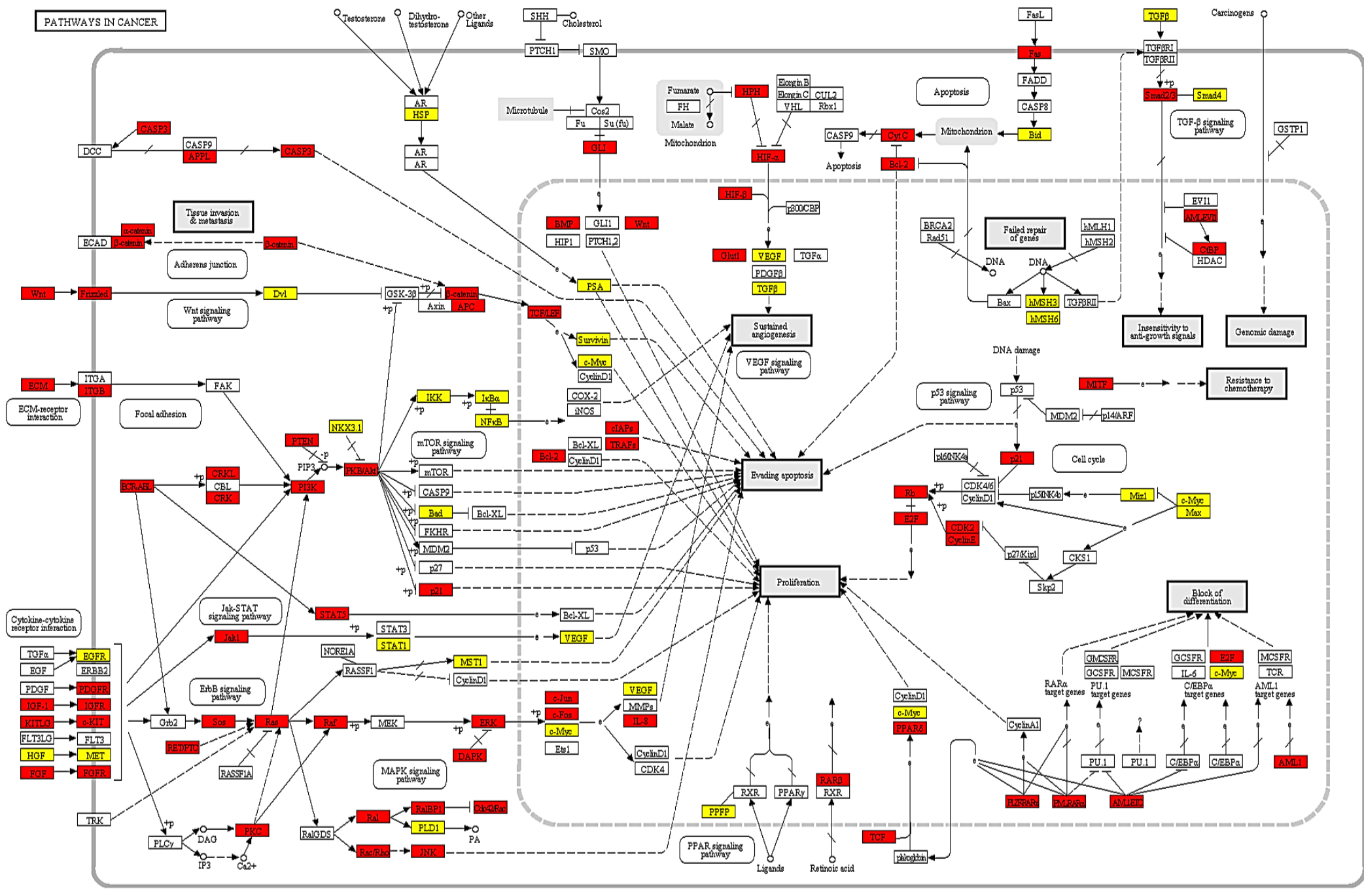

Supplement: Additional file 6 — Figure S3. KEGG “Pathways in cancer”: 68 targets of 10 miRNAs are involved in the pathway (red boxes). 38 genes targeted by the other miRNAs are colored in yellow; and 62 genes involved in the pathway were excluded from the BayMiR target list since their expression variabilities across arrays were very low (white boxes). The miRNA family IDs: miR-17/17-5p/20ab/20b-5p/93/106ab/427/518a-3p/519d,miR-548ah/3609,miR-4729,miR-203,miR-548p,miR-3647-3p,miR-300/381/539-3p,miR-142-5p,miR-545,miR-125a-5p/125b-5p/351/670/4319’. [file 1471-2164-14-592-S6.pdf]

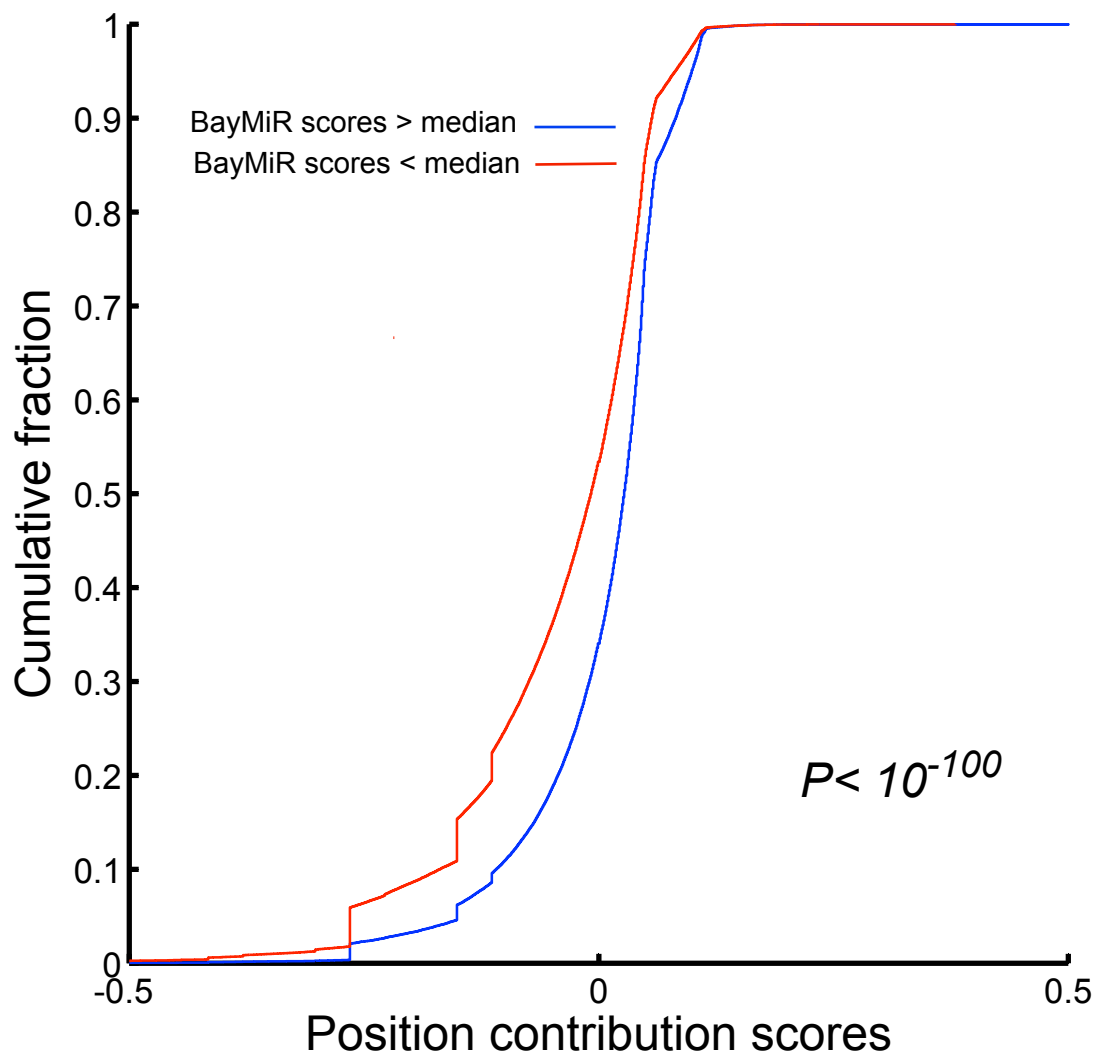

Supplement: Additional file 8 — Figure S7. Blue: the position contribution scores of miRNA-mRNA pairs whose BayMiR scores > median B a y M i R s c o r e s . Red: the position contribution scores of miRNA-mRNA pairs whose BayMiR scores < median B a y M i R s c o r e s . [file 1471-2164-14-592-S8.pdf]

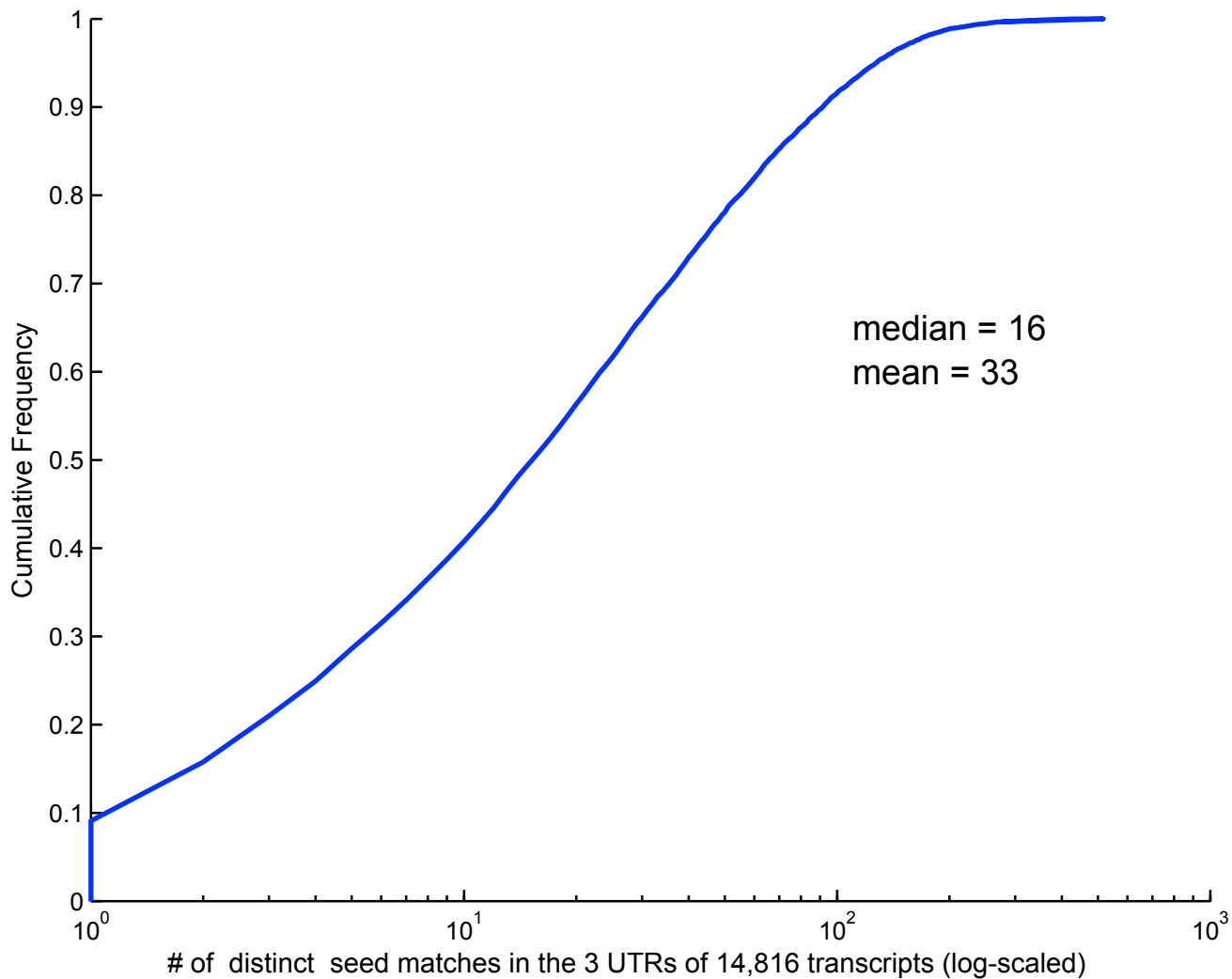

Supplement: Additional file 9 — Figure S4. The 3' UTR of mRNAs harbor many conserved seed matches. Shown is the cumulative distribution of number of seed matches in the 3'UTR of 14,816 mRNA transcripts with at least one miRNA seed match. [file 1471-2164-14-592-S9.pdf]

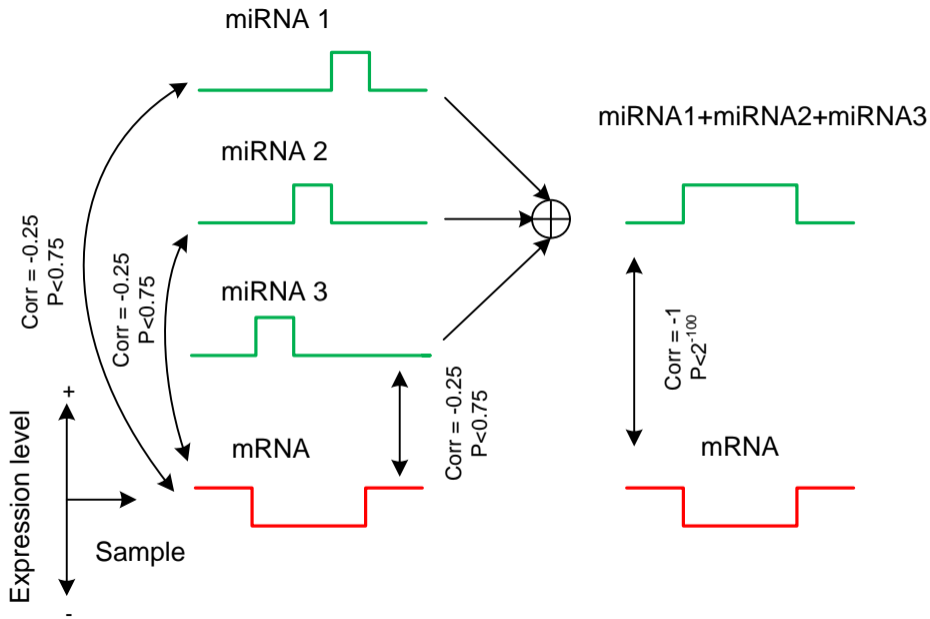

Supplement: Additional file 10 — Figure S5. Example of combinatorial regulation masking inverse correlation. Shown in green is the expression level of a target gene and in red the expression levels of three targeting miRNAs. The negative correlation of each individual miRNAs with the target is insignificant, but when considered together they explain perfectly the down-regulation impact of miRNAs. [file 1471-2164-14-592-S10.pdf]

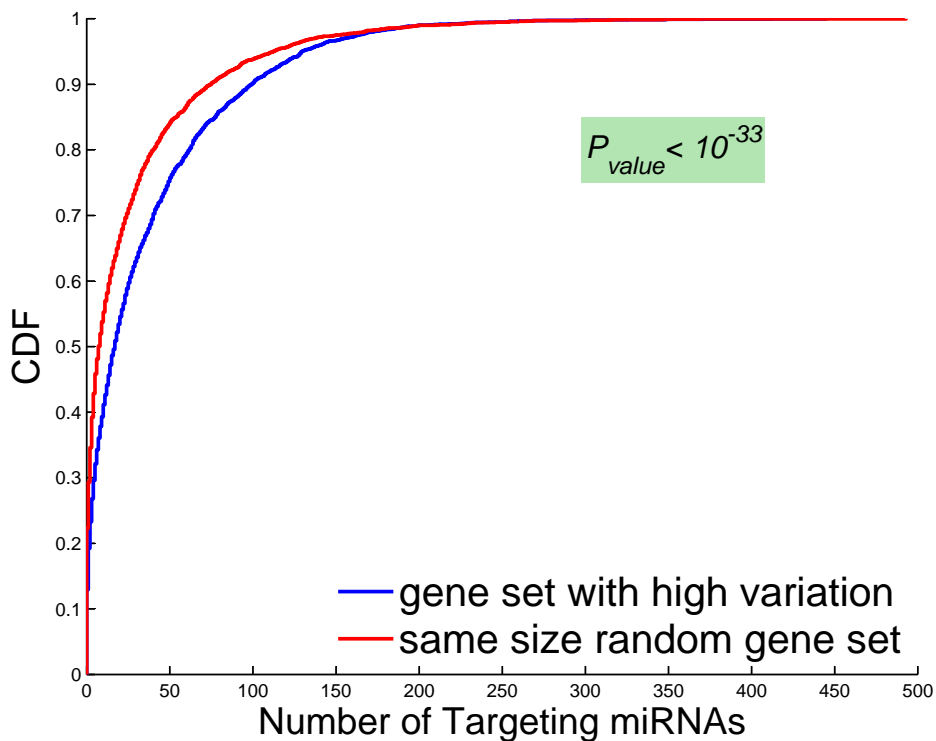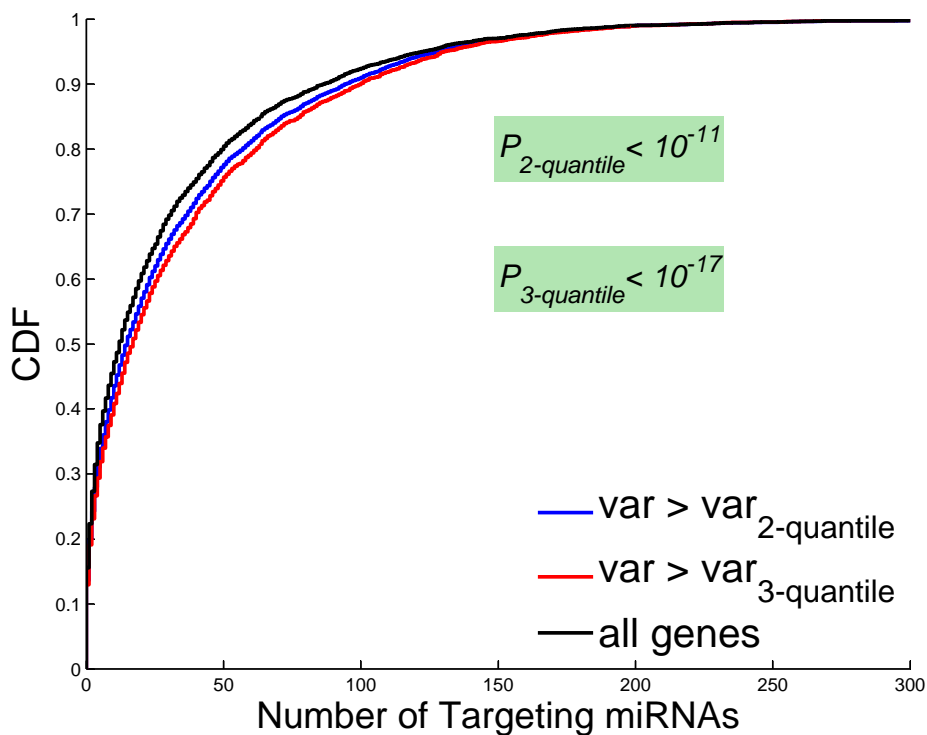

Supplement: Additional file 11 — Figure S6. Gene expression variability increases as the number of target sites increases in the 3’ UTR of genes. (top) miRNA targets have high expression variation. (bottom) Red and blue demonstrate the cumulative distributions of genes whose variance is larger than median and 75th percentile, respectively. Dark: cumulative distribution of variances corresponding to all genes. [file 1471-2164-14-592-S11.pdf]
