# Supplementary material for: BayMiR: inferring evidence for endogenous miRNA-induced gene repression from mRNA expression profiles
Source: BMC Genomics. 2013 Aug 30;14:592. doi: 10.1186/1471-2164-14-592 (PMC3933272; doi:10.1186/1471-2164-14-592)
Supplement: Additional file 5 — Table S3. Validated KEGG pathways. List of miRNAs with proposed functions found in our enriched KEGG list; the third column gives the Pubmed IDs of the references. [file 1471-2164-14-592-S5.pdf]

miRNAs	Pathways	PMID
miR-17/20ab/93/106ab/519d	Pathways in cancer	16461460;18485879;
miR-17/20ab/93/106ab/519d	Pathways in cancer	18328430;20101220;
miR-124/124ab/506	Axon guidance	18619591;
miR-138/138ab	Pathways in cancer	18201269; 20332227;
miR-155	T-cell-receptor signaling	17463289;19877012;
miR-17/20ab/93/106ab/519d	Pathways in cancer	18596939;19135980;
miR-15abc16abc/195/424/497	p53 signaling pathway	19626115;
miR-17/20ab/93/106ab/519d	Pathways in cancer	17608773;19066217;
miR-17/20ab/93/106ab/519d	MAPK signaling pathwa	18700987;
miR-17/20ab/93/106ab/519d	p53 signaling pathway	19696742;
miR-200bc/429/548a	Pathways in cancer	19671845;18829540;
miR-200bc/429/548a	Pathways in cancer	17804704;18376396;
miR-1ab/206/613	Pathways in cancer	18593897;19684618 ;
miR-17/20ab/93/106ab/519d	Pathways in cancer	19597473;16461460;
miR-25/32/92abc/363/367	Phosphoinositide signal	20388916;
miR-302abcde/372/373/520	Pathways in cancer	17695719;18193036;
miR-29abcd	Pathways in cancer	19247375;19818597;
miR-29abcd	Focal adhesion	19956414;
let-7/98/4458/4500	bladder cancer	21993544;
miR-29abcd	Small cell lung cancer	17890317;
miR-302abcde/372/373/520	Cell cycle	18328430;
miR-133abc	Apoptosis	17715156;
miR-17/20ab/93/106ab/519d	Cell cycle	18700987;
